# HPLC-DAD analysis, antinociceptive and anti-inflammatory properties of the ethanolic extract of Hyptis umbrosa in mice

**DOI:** 10.17179/excli2016-698

**Published:** 2017-01-02

**Authors:** Klécia S. dos Anjos, Heitor G. Araújo-Filho, Marcelo C. Duarte, Vicente C.O. Costa, Josean F. Tavares, Marcelo S. Silva, Jackson R.G.S. Almeida, Nathália A.C. Souza, Larissa A. Rolim, Irwin R.A. Menezes, Henrique D.M. Coutinho, Jullyana S.S. Quintans, Lucindo J. Quintans-Júnior

**Affiliations:** 1 Department of Physiology. Laboratory of Neurosciences and Pharmacological Assays Federal University of Sergipe, São Cristóvão, Sergipe, Brazil; 2 Federal University of Paraíba, João Pessoa, Paraíba, Brazil; 3 Federal University of San Francisco Valley, Petrolina, Pernambuco, Brazil; 4 Analytical Center of Drugs, Medicines and Food - CAFMA. Federal University of San Francisco Valley, Petrolina, Pernambuco, Brazil; 5 Department of Biological Chemistry. Regional University of Cariri, Crato, CE, Brazil

**Keywords:** medicinal plants, phenolic compounds, nociception, inflammatory pain

## Abstract

*Hyptis umbrosa* (syn. *Mesosphaerum sidifolium*) (Lamiaceae Family) has been used to treat several conditions such as gastrointestinal disorders, skin infections, nasal congestion, fever and cramps. The objective of this study was to evaluate the chemical composition, analgesic and anti-inflammatory profiles of ethanol extract from leaves of *Hyptis umbrosa* (EEB). HPLC-DAD was used to determine the fingerprint chromatogram of the extract. Male Swiss mice were orally pretreated with EEB (100, 200 or 400 mg/kg; 60 min before initiating algesic stimulation) and antinociceptive activity was assessed using the acetic acid-induced writhing model, formalin test and hyperalgesia induced by glutamate or capsaicin. Also, peritonitis was induced by the intrathoracic injection of carrageenan to quantify the total number of leukocytes. The presence of phenolic compounds in the extract was confirmed using HPLC-DAD. The treatment with EEB, at all doses, produced a significant analgesic effect against acetic acid-induced antinociceptive activity. In the formalin test, only the 400-mg/kg-dose of EEB had a significant effect in the first phase. However, all doses tested were able to reverse nociception in the second phase. The effect of all doses of EEB also showed a significant antinociceptive effect in the glutamate and capsaicin tests and inhibited the carrageenan-induced leukocyte migration to the peritoneal cavity. The present study suggests that the EEB possesses peripheral analgesic action and showed potential in reducing the spreading of the inflammatory processes. Also, it seems to be related with vanilloid and glutamate receptors.

## Introduction

The species of Lamiaceae are composed of several species, such as *Hyptis pectinata*,* Hyptis mutabilis*, *Hyptis fruticosa* and *Hyptis umbrosa*. Such plants are frequently found on the northeastern Brazilian coast (Joly, 1998[19]). Plants of *Hyptis* species have great economical and ethnopharmacological importance (Franco et al., 2011[13]). Under the chemical point of view, there are compounds belonging to classes of metabolites, the acetate pathway, the shikimate pathway and also the compounds of mixed biosynthetic origin (Falcão and Menezes, 2003[9]; Franco et al., 2011[13]).

It has been alleged that the *Hyptis* genus possesses medicinal properties and it is recommended in folk medicine for the treatment of several conditions, such as gastrointestinal disorders, skin infections, nasal congestion, fever and cramps (Rojas et al., 1992[43]). Additionally, redox, analgesic and anti-inflammatory profiles have been reported to this genus (Franco et al., 2011[12]; Lima et al., 2013[25]; Menezes et al., 2015[31]; Paixão et al., 2015[35], 2013[36]). 

The* Hyptis umbrosa *is popularly known as “aleluia do serrote” (Silva and Andrade, 2004[47]) and “alfazema do mato” (Fernandes, 2007[10]). There are few reports in the literature on phytochemical and pharmacological studies of this species (Piozzi et al., 2009[38]). According to Matida et al. (1986[29]), the presence of ursolic acid and 2α-hidroxy-ursolic acid was already confirmed in the ethanolic extract from leaves of *H. umbrosa*. 

Fifty nine components of the essential oil from aerial parts of *Hyptis umbrosa* have already been identified through chromatographic methods. Among the major components found, terpenes were more highlighted, such as fenchone (24.8 %), cubebol (6.9 %) and limonene (5.4 %) (Rolim, 2013[44]). Such data can contribute to a better understanding of the pharmacological effects of the extract under study since the terpenes have been described for their analgesic and anti-inflammatory properties, mainly to acute and inflammatory pains (Guimarães et al., 2013[15], 2014[16]; Quintans-Júnior et al., 2013[41]; Quintans et al., 2014[39]; Siqueira-Lima et al., 2014[48], 2016[49], 2017[50]).

Due to the medicinal folk use of *Hyptis umbrosa*, mainly as tea, and lack of knowledge on its pharmacological properties, we evaluated antinociceptive and anti-inflammatory effects of the ethanol extract from leaves of *H. umbrosa* (EEB) in experimental protocols.

## Material and Methods

### Drugs and reagents

The drugs and reagents used in this study were morphine sulphate (Dimorf-Cristália, Brazil), glutamate (Sigma, USA), capsaicin (Sigma, USA), λ-Carrageenan (Sigma, USA), naloxone (Res. Biochemicals Inc., USA), dexamethasone (União Química, Brazil) and formaldehyde (Merck, USA). The vehicle was 0.2 % Tween 80 (Sigma, USA) dissolved in saline solution (NaCl 0.9 %).

These chemical reference substances were used: caffeic acid, chlorogenic acid, gallic acid, *p*-coumaric acid, protocatechuic acid, tannic acid, apigenin, borneol, catechin, chrysin, epicatechin, epigallocatechin, fisetin, gallocatechin, kaempferol, lupeol, myricetin, narigenin, quercetin, quercetin 3-β-glucoside, resveratrol, rutin and scopoletin (purity ≥ 97 %), which were obtained from Sigma Aldrich (USA). HPLC grade methanol was acquired by Carlo Erba (France). Acetonitrile of HPLC grade was purchased from Merck (Germany). HPLC-grade trifluoroacetic acid was obtained from Sigma (USA) with ultrapure water.

### Animals

Experimental protocols were performed using male Swiss mice (25-30 g) obtained from the Animal Facilities of the Federal University of Sergipe (UFS). Mice were housed in controlled-temperature rooms (21 ± 2°C), under a 12 h light/dark cycle, with access to water and food *ad libitum* until use. Experimental protocols were approved by the Animal Care and Use Committee at UFS/Brazil (CEPA # 47/09). Nociceptive and inflammatory tests were performed under blind conditions and all efforts were made to minimize the number of animals used and their discomfort.

### Sample preparation 

The leaves of *H. umbrosa* were collected in the municipality of Matureia (7° 11′ 10′′ S, 37° 08′ 22′′ and 37° 25′ 53′′ W), State of Paraíba, in July 2009. The plant material was identified by Prof. Maria de Fátima Agra, Head of the Botany Section of the Pharmaceutical Technology Laboratory (Laboratório de Tecnologia Farmacêutica, LTF). A voucher specimen (AGRA 5541) is deposited at the Prof. Lauro Pires Xavier herbarium (JPB) of the Federal University of Paraíba. Dried leaves (2 kg) were exhaustively extracted with 95 % EtOH at room temperature for three days. The extract obtained was concentrated in a rotary evaporator under reduced pressure at 40° C, yielding 200.0 g of EEB.

A solution of 1 mg/mL of the *H. umbrosa* extract was prepared with HPLC-grade methanol, which was sonicated until complete solubilization. The solution was filtered through a membrane filter of 0.22 μM pore (Chromafil^®^ Xtra, USA).

### HPLC conditions

The chromatographic analyses were performed on a HPLC from Shimadzu^®^ with a diode array detector (DAD) and a C_18_ column with dimensions of 250 x 4.6 mm, 5 μm (Hypersil ThermoScientific^®^) with guard column, and the temperature was kept stable at 30° C throughout the analysis. Two solutions were used as mobile phase: Solution A consisted of ultrapurified water + trifluoroacetic acid 0.1 % (v/v) and B, acetonitrile solution. Samples were analyzed using a gradient system described in the Table 1(Tab. 1), with a 0.6 mL.min^-1^ flow.

The analytical standards and samples were injected in the volume of 30 μL and the detection was performed in DAD at a wavelength of 270 nm. The software LCSolution 1.0 (Shimadzu^®^, Japan) was used for the data analysis.

### Pharmacological assessment

#### Acetic acid-induced abdominal writhes

Abdominal writhes consisted of a contraction of the abdominal muscle together with a stretching of the hind limbs, induced by intraperitoneal (i.p.) injection of acetic acid (0.75 % solution, 0.1 mL/10 g) in mice, the algogen agent (Koster et al., 1959[22]). The animals were previously treated via oral route (gavage, p.o.) with different doses of EEB (100, 200 or 400 mg/kg) 60 min before initiating algesic stimulation, or with morphine (MOR, 5 mg/kg, as positive control), in the absence and presence of naloxone (NAL) (n = 8/per group). The control group received vehicle, saline + 0.2 % Tween 80 (0.1 mL/10 g, p.o., 60 min beforehand, n = 8, per group). The abdominal writhes were registered, in separate individual chambers, for a period of 20 min (for each animal), starting after the administration of acetic acid.

#### Formalin test

The formalin test was applied according to the method of Hunskaar and Hole (1987[18]). Mice were pre-treated with EEB (100, 200, or 400 mg/kg, p.o., 60 min beforehand), or with morphine (MOR, 5 mg/kg; i.p., 30 min beforehand), in the absence and presence of naloxone (NAL), before intraplantar injection of 1 % formalin solution (20 µL) into the right hind paw of the animal (n = 8 per group). The control group received vehicle, similar to the previous test (writhing test). The time during which the animal spent licking or biting its paw was measured during the first-phase (0-5 min) and the second-phase (15-30 min) of the test.

#### Glutamate- and capsaicin-induced nociception

The methods used were similar to that described previously (Quintans-Junior et al., 2010[42]). The mice were placed individually for 5 min in a transparent plexiglass cage observation chamber (25 cm × 15 cm × 15 cm) as an adaptation period. After that, either 20 μmol glutamate per paw or 20 μL of capsaicin (1.6 μg/paw prepared in a phosphate-buffered solution) was injected under the skin of the dorsal surface on the right hind paw. The mice were pre-treated with EEB (100, 200, or 400 mg/kg, p.o.) or morphine (MOR, 5 mg/kg) 1 h before the injection of the irritant agent (glutamate or capsaicin). The control animals received a similar vehicle of the early tests. The amount of time spent licking the injected paw was timed with a chronometer and was considered indicative of nociception.

#### Carrageenan-induced peritonitis

Peritonitis was induced by the intrathoracic (i.t.) injection of carrageenan (500 µg/ cavity, 500 µL, i.p) diluted in sterile saline. Control animals received the same volume of vehicle. The animals were pretreated with EEB (100, 200, or 400 mg/kg, p.o.), dexamethasone (2 mg/kg, s.c.) or vehicle (saline + 0.2 % Tween 80, p.o.) 60 min before the injection of carrageenan. Four hours after stimulation, the animals were sacrificed in a CO_2_ chamber; the pleural cavities were opened and washed with 1 mL of PBS (1×) containing EDTA (10 mM). Total leukocyte counts collected in the pleural lavage were performed on a Neubauer chamber under an optical microscope. The samples were diluted (40×) in Türk solution.

### Statistical analysis

Values are expressed as mean ± SEM. The data obtained were evaluated through the one-way analysis of variance (ANOVA) followed by Tukey's test. In all cases, differences were considered significant if p > 0.05. All statistical analyses were performed using the software GraphPad Prism 5.0 (GraphPad Prism Software Inc., San Diego, CA, USA).

## Results

### HPLC Analysis

**Figure 1:**
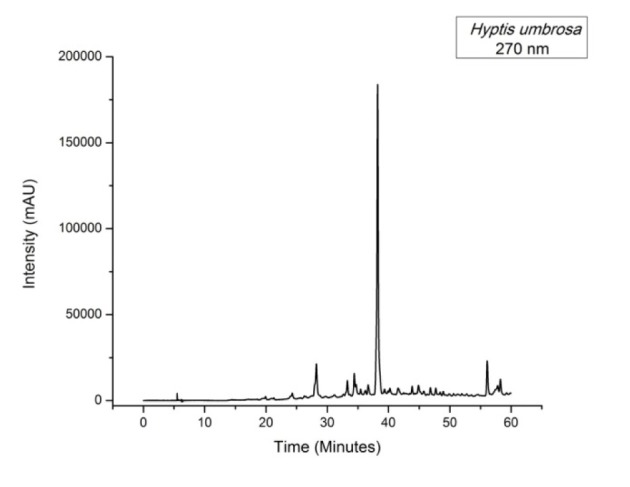
Figure 1: Chromatogram of *H. umbrosa* ethanolic extract (270 nm)

In this study we developed a method based on HPLC-DAD in order to obtain a chromatographic system that was able to elute and provide good resolution in the separation of compounds in the *H. umbrosa* extract, as it can be seen in Figure 1(Fig. 1).

By analyzing the chromatographic profile of the sample, one can observe the presence of a major compound at retention time 38.25 min, which showed an absorption spectrum λ 206/318 (Figure 1(Fig. 1)) with 98 % purity.

It was not possible to identify the substances present in the *H. umbrosa* extract through the comparison of retention time and maximum absorption spectra at the analytical standards (Table 2(Tab. 2)). Therefore, no similarity to any of the evaluated analytical standards was found. These compounds are under investigation.

### Effect of EEB on the acetic-acid-induced writhing test in mice

**Figure 2:**
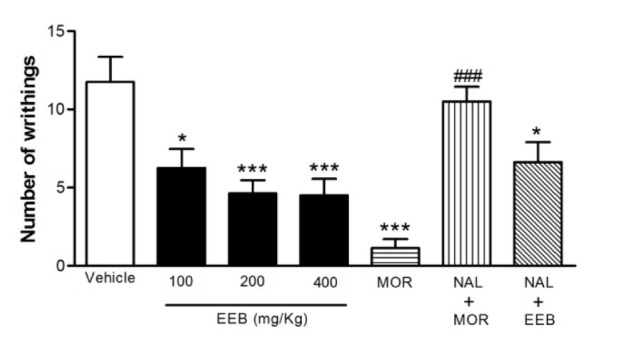
Effect of EEB of *H. umbrosa* on the acetic acid-induced writhing test in the absence and presence of naloxone in mice. Vehicle (control), EEB (100, 200 and 400 mg/kg) or morphine (MOR) were administered p.o. 1 h before acetic acid injection. Pre-treatment with naloxone (NAL, 1.5 mg/kg, i.p.) was performed 0.5 h before treatment (i.p.) with EEB (400 mg/kg), or MOR (3 mg/kg). Each column represents mean ± S.E.M. (n = 6, per group). *p < 0.05 or ***p < 0.001 *versus* control. ###p < 0.001 *versus* MOR group (ANOVA followed by Tukey's test).

The results shown in Figure 2(Fig. 2) demonstrated that the pretreatment with EEB (100, 200, or 400 mg/kg, p.o.) was capable of inhibiting the abdominal writhing induced by the intraperitoneal administration of the acetic acid when compared with the control group (p < 0.05 or p < 0.01). Morphine (3 mg/kg), an opioid agonist, was used as positive control group and significantly (p < 0.001) suppressed the acetic acid-response. 

Pretreatment with NAL (1.5 mg/kg, i.p.), an opioid antagonist, was performed 0.5 h before treatment (i.p.) with EEB (400 mg/kg) or MOR (3 mg/kg) and it was observed that naloxone significantly (p < 0.001) inhibited the antinociceptive effect of the morphine. However, naloxone was incapable of reversing the action of EEB (400 mg/kg) when compared with the control group (Figure 2(Fig. 2)).

### Effect of EEB on the formalin-induced nociception in mice

**Figure 3:**
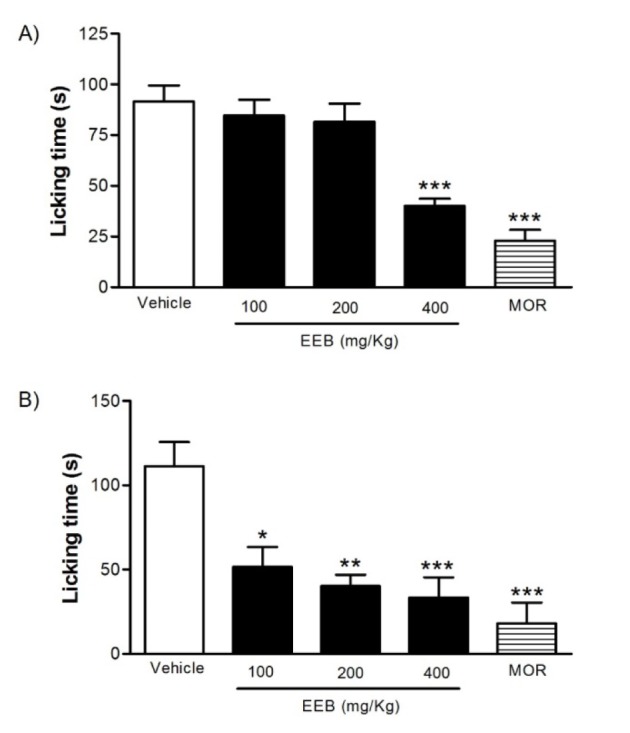
Effects of EEB of *H. umbrosa* on the formalin-induced nociception in mice. Vehicle (control), EEB (100, 200 and 400 mg/kg) or morphine (MOR) were administered p.o. 1 h before formalin injection. (A) Represents the first phase and (B) represents second phase of formalin-induced nociception. Each column represents mean ± S.E.M. (n = 8, per group). *p < 0.01, ** p > 0.01 or ***p < 0.001 versus control (ANOVA followed by Tukey's test).

The pretreatment with EEB (100, 200, or 400 mg/kg, p.o.) had significant effect (p < 0.001) only at the highest dose during the first phase of the test (0-5 min), whereas during the second phase (20-25 min), EEB caused a significant reduction in the licking time in all doses tested when compared to the control group (Figure 3(Fig. 3)). Morphine was capable to inhibit both phases of the pain stimulus (p < 0.001).

### Effect of EEB on the glutamate-induced nociception in mice

**Figure 4:**
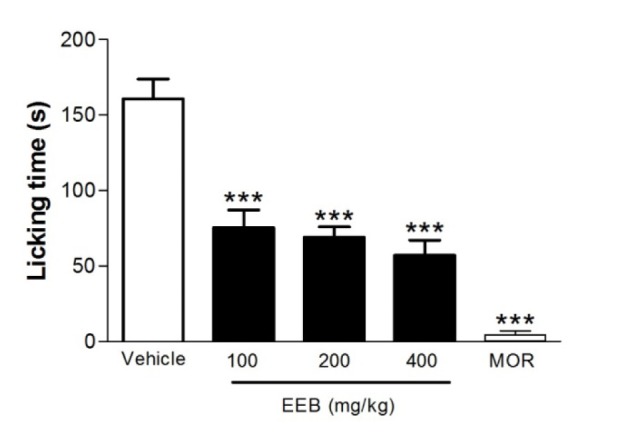
Effects of EEB of *H. umbrosa* on the glutamate-induced nociception in mice. Vehicle (control), EEB (100, 200 and 400 mg/kg) or morphine (MOR) were administered p.o. 1 h before glutamate injection. Each column represents mean ± S.E.M. (n = 8, per group). ***p < 0.001 versus control (ANOVA followed by Tukey's test).

The results of the nociception induced by the glutamate test are represented in Figure 4(Fig. 4). Pretreatment with EEB (100, 200, or 400 mg/kg, p.o.), as well as MOR, significantly decreased the face-rubbing behavior compared with the control group (p < 0.001).

### Effect of EEB on the capsaicin-induced nociception in mice

**Figure 5:**
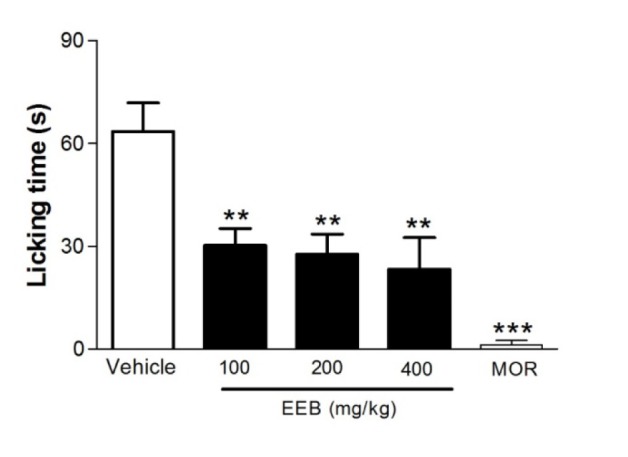
Effects of EEB of *H. umbrosa* on the capsaicin-induced nociception in mice. Vehicle (control), EEB (100, 200 and 400 mg/kg) or morphine (MOR) were administered p.o. 1 h before capsaicin injection. Each column represents mean ± S.E.M. (n = 8, per group). **p < 0.01 or ***p < 0.001 versus control (ANOVA followed by Tukey's test).

Figure 5(Fig. 5) shows that EEB significantly (p < 0.01) reduced the face-rubbing behavior induced by the administration of capsaicin. Morphine also decreased significantly (p < 0.001) the orofacial behavior compared with the control group.

### Effect of EEB on CG-induced peritonitis

**Figure 6:**
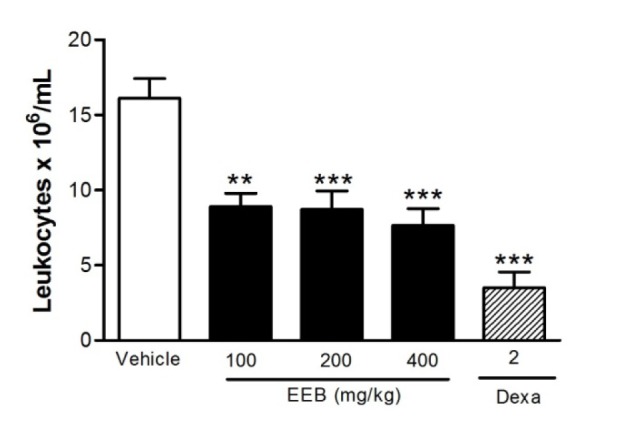
Figure 6: Effect of EEB of *H. umbrosa* on leukocyte migration into the peritoneal cavity induced by carrageenan in mice. Groups of rats were pre-treated with vehicle (control), dexamethasone (Dexa, 2 mg/kg, s.c.) or EEB (100, 200 and 400 mg/kg) 60 min before carrageenan (500 µg/cavity, 500 µL, i.p.)-induced peritonitis. Cell counts were performed at the time 4 h after the injection of carrageenan. Each value represents the mean ± S.E.M. **p < 0.01 or ***p < 0.001 related to control group. ANOVA followed by Tukey's test.

Figure 6(Fig. 6) shows the inhibitory effect of all doses of EEB on CG-induced response (p < 0.01 or p < 0.001). The results obtained with the control group support the effect of EEB since the vehicle presented no activity, and the control drug dexamethasone inhibited (p < 0.001) the carrageenan-induced leukocyte migration to the peritoneal cavity.

## Discussion

*H. umbrosa* is a medicinal plant used in the folk medicine of northeastern Brazil. However, little is known about its possible biological activities. Hence, this is the first report on the ethanol extract from leaves of *H. umbrosa *(EEB), by oral administration, as analgesic and anti-inflammatory agents using experimental protocols in mice. 

The acetic acid-induced writhing reaction in mice, described as a typical model for inflammatory pain, has long been used as a classic model for the assessment of analgesic or anti-inflammatory properties of new agents (Mohamad et al., 2010[33]). Acetic-acid causes tissue damage and acts indirectly by inducing the release of chemical mediators such as substance P, bradykinins, prostaglandins especially PGI_2_ as well as pro-inflammatory cytokines such as IL-1, IL-6, IL-8 and TNF-α which stimulate the nociceptive neurons that are sensitive to non-steroidal anti-inflammatory drugs (NSAIDs) and to opioids drugs (Collier et al., 1968[6]; Kumazawa et al., 1996[23]; Le Bars et al., 2001[24]; Ulugol et al., 2006[52]).

The results of the present study showed that all doses of EEB (100, 200 and 400 mg/ kg) had a significant effect on reducing the number of writhes when compared to the control group. The genus *Hyptis* (Lamiaceae) is composed of several plant species and many of them have already presented activity in inhibiting the writhing induced by acetic acid characterizing the peripheral effect of the genus. The administration of six genotypes of volatile oils of *H. pectinata* significantly reduced the number of writhes induced by acetic acid in a range from 62.5 % to 84.8 % (Arrigoni-Blank et al., 2008[2]). Similarly, Bispo et al. (2001[5]) demonstrated that the aqueous extract of *H. pectinata* exhibited the same effect of EEB when the dose of 200 mg/kg showed maximal effect on the number of writhes, since no significant difference was observed between the doses of 200 and 400 mg/kg. Providing further support of the antinociceptive potential of the genus, essential oil of the *H. fruticosa *also proved to have peripheral action in this model, inhibiting 35.7 % of writhes in its highest dose (Menezes et al., 2007[30]).

In order to better evaluate the possible central action of EEB, the animals were pre-treated with NAL (1.5 mg/kg, i.p.) 0.5 h before treatment (i.p.) with EEB (400 mg/kg) or MOR (3 mg/kg). The data suggest that NAL was able to antagonize the action of MOR (p < 0.001); however, it was unable to reverse the antinociceptive action of EEB. 

The analgesic mechanism of action of EEB can, probably, involve inhibition of the synthesis and/or release of inflammatory mediators which promote pain in the nociceptive nervous terminations, similarly to the NSAIDs suggesting a peripheral analgesic action. However, this method shows good sensitivity but is non-specific, since NSAIDs, opioid analgesics and even tricyclic antidepressants may inhibit the nociceptive response in this model (Dai et al., 2002[7]; Franca et al., 2001[11]; Gonzalez et al., 2001[14]). Additionally, plants of other species, such as *H. pectinata*, were able to show a possible opioid action when antagonized by naloxone; however, a more specific test (hot plate) was used, known to assess the central action of new drugs (Arrigoni-Blank et al., 2008[2]; Lisboa et al., 2006[26]).

In an attempt to obtain further evidence about the analgesic effect of EEB, we performed the formalin test in mice. The formalin test is a widely used test which helps to elucidate the mechanism of action of the drug under study (Le Bars et al., 2001[24]). This test involves two phases: the first phase (neurogenic phase) results essentially from the direct stimulation of nociceptors and the second phase (inflammatory phase) which is originated from peripheral mechanisms with the release of chemical mediators (serotonin, histamine, bradykinin and prostaglandins (Le Bars et al., 2001[24]; Murray et al., 1988[34]; Tjolsen et al., 1992[51]). Furthermore, the two phases of this test are sensitive to opioid analgesics whereas NSAIDs seem to suppress only the second phase (Hunskaar and Hole, 1987[18]; Malmberg and Yaksh, 1992[28]; Shibata et al., 1989[46]).

Now, we demonstrated that all doses of EBB tested significantly decreased the nociceptive response of the second stage, whereas only the highest dose was successful in the first phase of the test. Knowing that chemical mediators such as serotonin, histamine, bradykinin, prostaglandins and nitric oxide are involved in the inflammatory phase of the test (Luccarini et al., 2006[27]), we can confirm that this compound has an anti-inflammatory effect further emphasizing its peripheral analgesic action.

Recently, plants of the genus *Hyptis* such as *H. pectinata* have showed an improvement in the analgesic and anti-inflammatory profile in the formalin test when complexed with β-cyclodextrin (Menezes et al., 2015[31]). These results can be attributed to the oil major component (*E*)-caryophyllene, a sesquiterpene that can exert analgesic profile through its capacity to modulate inflammatory and neuropathic pain (Klauke et al., 2014[21]; Quintans-Júnior et al., 2016[40]).

There is ample evidence that the excitatory amino acids are responsible for the transmission of sensory information originating in the periphery to the CNS. Moreover, it is well established that glutamate injection evoked pronounced nociceptive responses, which are mediated by the activation of cellular mediators and activatation of glutamate receptors (e.g. NMDA) (Beirith et al., 2002[3]). These processes combined with other pro-inflammatory mediators and cytokines (TNF-α and IL-1β) act synergistically in neuronal excitability (Bernardino et al., 2005[4]; Millan, 1999[32]).

Pain signals induced by glutamate could be associated with peripheral, spinal and supraspinal influences through mediation by NMDA and non-NMDA receptors (Beirith et al., 2002[3]). Since all doses of EEB exhibited an inhibition of the nociceptive behavior (p < 0.001) on glutamate-induced nociception, it can be suggested that it interferes with the glutamatergic system. 

Capsaicin-induced pain model is used to test drugs with neurogenic origin (Sakurada et al., 2003[45]). Capsaicin is sensitive to transient receptor potential vaniloid 1 (TRPV1) and plays an important role in pain transduction through the Ca^+2^ influx channels and release of neuropeptides (substance P), excitatory amino acids (glutamate and aspartate), nitric oxide and other pro-inflammatory substances from peripheral terminals (Honda et al., 2008[17]; Pelissier et al., 2002[37]; Vogt-Eisele et al., 2007[53]). In this respect, our results showed that EEB was able to reduce behavioral response of the capsaicin-induced nociception (p < 0.01), suggesting a possible modulation of the compound in the TRPV1 receptors.

Considering that leukocyte migration is a dynamic process involving the activation and release of inflammatory cytokines and chemical mediators that synergistically promote the excitability of nociceptors (Adams and Nash, 1996[1]; Dray, 1995[8]; Kasama et al., 2005[20]), we assessed the leukocyte migration through carrageenan-induced peritonitis and observed that the EEB had an inhibitory effect on leukocyte migration further proving the activity of this compound in inflammatory pain.

## Conclusions

In conclusion, the present study suggests that the EEB possesses peripheral analgesic action possibly mediating the release of chemical agents and also showed potential in reducing the spreading of the inflammatory processes by acting in leukocyte migration. The mechanism of action exerted by EEB is currently under investigation, but it seems to be related with vanilloid and glutamate receptors.

## Notes

Jullyana S.S. Quintans and Lucindo J. Quintans-Júnior (Laboratory of Neurosciences and Pharmacological Assays, Federal University of Sergipe, Av. Marechal Rondom, São Cristóvão, Sergipe-Brazil; Tel.: +55-79-21056645, fax: +55-79-3212-6640, E-mail: lucindojr@gmail.com) contributed equally as corresponding authors.

## Conflict of interests

The authors declare that there is no conflict of interests regarding the publication of this paper.

## Funding

The authors were supported by grants from FAPITEC-SE, CAPES, CNPq and FINEP, all from Brazil.

## Acknowledgements

Authors thank Jessica Deise Santos Dias (*in memoriam*) for technical assistance. We thank teacher Abilio Borghi for the grammar review of the manuscript.

## Figures and Tables

**Table 1:**
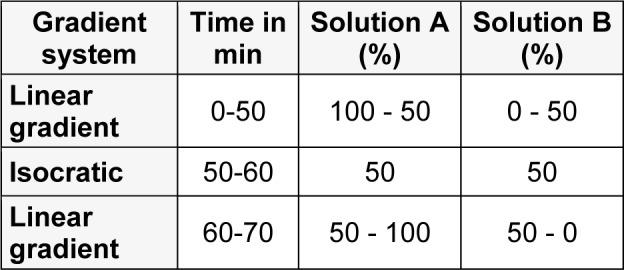
Table 1: Gradient system used in the analysis through HPLC-DAD

**Table 2:**
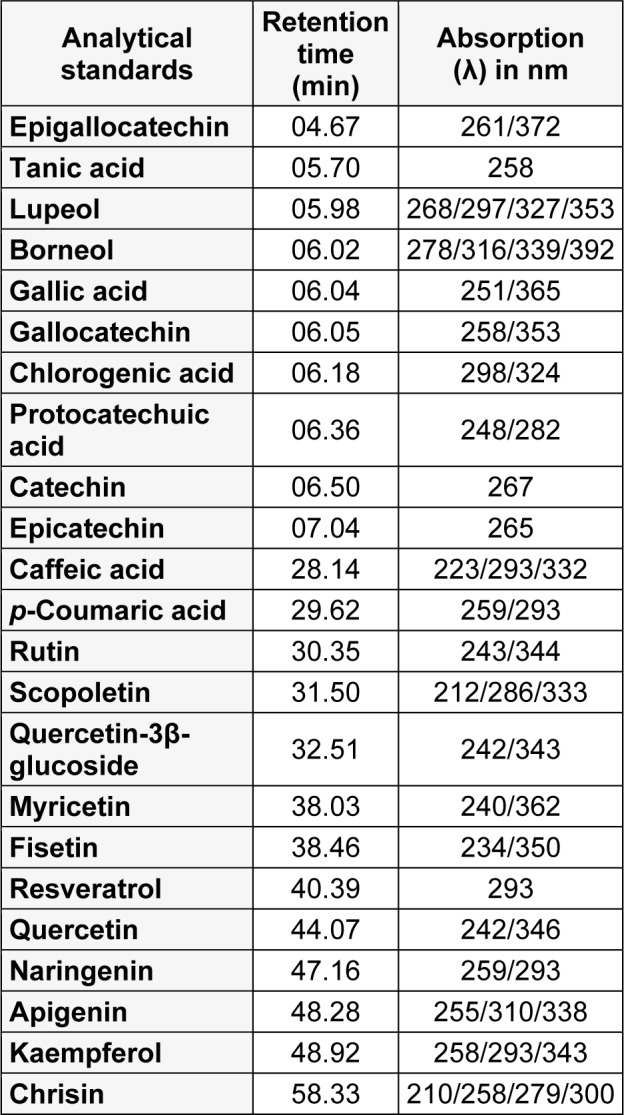
Results obtained after the analysis of the analytical standards
